# GTRD: a database of transcription factor binding sites identified by ChIP-seq experiments

**DOI:** 10.1093/nar/gkw951

**Published:** 2016-10-24

**Authors:** Ivan Yevshin, Ruslan Sharipov, Tagir Valeev, Alexander Kel, Fedor Kolpakov

**Affiliations:** 1 BIOSOFT.RU, LLC, Novosibirsk 630058, Russian Federation; 2 Institute of Computational Technologies SB RAS, Novosibirsk 630090, Russian Federation; 3 Novosibirsk State University, Novosibirsk 630090, Russian Federation; 4 A.P. Ershov Institute of Informatics Systems SB RAS, Novosibirsk 630090, Russian Federation; 5 Institute of Chemical Biology and Fundamental Medicine SB RAS, Novosibirsk 630090, Russian Federation

## Abstract

GTRD—Gene Transcription Regulation Database (http://gtrd.biouml.org)—is a database of transcription factor binding sites (TFBSs) identified by ChIP-seq experiments for human and mouse. Raw ChIP-seq data were obtained from ENCODE and SRA and uniformly processed: (i) reads were aligned using Bowtie2; (ii) ChIP-seq peaks were called using peak callers MACS, SISSRs, GEM and PICS; (iii) peaks for the same factor and peak callers, but different experiment conditions (cell line, treatment, etc.), were merged into clusters; (iv) such clusters for different peak callers were merged into metaclusters that were considered as non-redundant sets of TFBSs. In addition to information on location in genome, the sets contain structured information about cell lines and experimental conditions extracted from descriptions of corresponding ChIP-seq experiments. A web interface to access GTRD was developed using the BioUML platform. It provides: (i) browsing and displaying information; (ii) advanced search possibilities, e.g. search of TFBSs near the specified gene or search of all genes potentially regulated by a specified transcription factor; (iii) integrated genome browser that provides visualization of the GTRD data: read alignments, peaks, clusters, metaclusters and information about gene structures from the Ensembl database and binding sites predicted using position weight matrices from the HOCOMOCO database.

## INTRODUCTION

Recognition of transcription factor (TF) binding sites (TFBSs) in genomes has been one of the most important tasks of modern biology since the introduction of the DNA footprint technique in 1978 (1). The progress in that field had been limited by the absence of massive high-throughput technology to permit the identification of DNA–protein interactions. The appearance of ChIP-seq technology developed independently by three research groups in 2007 (2–4) allowed this hurdle to be overcome. This achievement resulted in an explosion in the number of freely available ChIP-seq datasets performed for different species, tissues and cell lines several years later. The well-known research project ENCODE selected ChIP-seq as one of the main assays to identify functional genomic elements starting from the phase II period (5). That decision permitted to improve the technology, related standards and pipelines for data processing, and made ChIP-seq popular worldwide. Several other variants of immunoprecipitation assay are also available, including ChIP-chip (6), ChIP-exo (7), ChIA-PET (8), etc., but ChIP-seq remains the most popular.

The accumulation of a large number of ChIP-seq datasets worldwide has led to the establishment of dedicated databases. There are currently several freely available databases indexing mainly ChIP-seq data (Table 1) oriented to TF binding motifs/sites. Only some of them contain data processed uniformly by their own workflows starting from raw data and ending with the identification of TFBSs. This aspect is quite important due to the differing quality of raw data obtained from various sources, conditions of experiments, abilities of applied algorithms, etc. None of these reported databases integrates data from different ChIP-seq experiments to provide non-redundant sets of TFBSs. Taking into account the shortcomings mentioned above and having a novel view of how such data and data processing should be organized, we have established a Gene Transcription Regulation Database (GTRD; http://gtrd.biouml.org). GTRD is integrated with a comprehensive software platform BioUML (http://www.biouml.org) with a wide spectrum of implemented methods for bioinformatics and systems biology. In this article we present the GTRD database that provides:

- comprehensive index of human and mouse ChIP-seq data from third-party sources;
- the most exhaustive catalog of ChIP-seq peaks for 476 human and 257 mouse TFs;
- non-redundant sets of TFBSs produced by a new metacluster approach based on the merging of different ChIP-seq experiments and results of different peak callers.

**Table 1.** Comparison of databases that are based on ChIP-seq data

Database, URL	Source of human and mouse data	Number of samples (TF-related)*	Number of TFs	Number of ChIP-seq peak callers used	Metaclus-ter approach	Uniform data processing	Genome browser
ChIPBase (http://rna.sysu.edu.cn/chipbase)	GEO, ENCODE	total 3549 human 2498 mouse 1036 rat 15	252 TFs and non-TFs for 10 species	>10 in total, but no uniform pipeline, each ChIP-seq is processed by different peak caller	No	No	Self-developed: deepView genomeView
Cistrome DB (http://dc2.cistrome.org/#/)	GEO, SRA, ENA, ENCODE	total 10 276 (TF+non-TF) human 5774 mouse 4502 rat 0	260 TFs and non-TFs	1 (MACS2)	No	Yes	UCSC genome browser
ENCODE (https://www.encodeproject.org)	ENCODE	total 1448 human 1254 mouse 194 rat 0	295 TFs and non-TFs for human, 52 TFs and non-TFs for mouse	5 (SPP, GEM, PeakSeq, MACS, Hotspot/Hotspot2)	No	Yes	Self-developed: UCSC genome browser and WashU epigenome browser
Factorbook (http://www.factorbook.org)	ENCODE	total 1007 human 837 mouse 170 rat 0	167 TFs, co-factors and chromatin remodeling factors for human, 51—for mouse	None	No	No	No
GTRD (http://gtrd.biouml.org)	GEO, SRA, ENCODE	total 5078 human 2955 mouse 2107 rat 16	476 human and 257 mouse sequence specific TFs, corresponding to 542 TFClass classes.	4 (MACS, SISSRs, GEM, PICS)	Yes	Yes	Self-developed
ChIP-Atlas (http://chip-atlas.org)	SRA	total 10 774 human 5914 mouse 4860 rat 0	699 human and 502 mouse TFs and others.	1(MACS2)	No	Yes	IGV
GeneProf (http://www.geneprof.org)	SRA, ENCODE, literature	total 1692 human 693 mouse 999 rat 0	133 human and 131 mouse TFs	1(MACS)	No	Yes	Self-developed: based on GenomeGraphs
NGS-QC (http://www.ngs-qc.org)	GEO	total 6672 human 4234 mouse 2438 rat 0	unknown	None	No	Yes	No

*The number of ChIP-seq samples cannot be directly compared between databases as definition of sample may be distinct.

## MATERIALS AND METHODS

### Data collection

ChIP-seq data for GTRD have been collected systematically from the following well-known public repositories: Sequence Read Archive (SRA; http://www.ncbi.nlm.nih.gov/sra) (9), ENCODE (https://www.encodeproject.org) (5), Gene Expression Omnibus (GEO; https://www.ncbi.nlm.nih.gov/geo/) (10) and literature, as well. Two main types of data have been collected:

- raw data in either FASTQ or SRA formats;
- metadata describing ChIP-seq experiments—information about target TF, cell source, used antibody, experimental conditions and control experiment.

In GTRD we include ChIP-seqs for sequence-specific TFs only. Since the definition of TF may vary, we have restricted GTRD to the factors presented in the TFClass database (http://tfclass.bioinf.med.uni-goettingen.de/tfclass) (11).

The GTRD processing pipeline starts with automatic querying of GEO and ENCODE for ChIP-seq experiment information. The GEO database contains ChIP-seq experiment descriptions in human-readable format that imposes some difficulties for automatic processing of large volumes of data. GEO was queried for ChIP-seq experiments programmatically using Entrez Programming Utilities (http://www.ncbi.nlm.nih.gov/books/NBK25501). Resulting GEO entries were downloaded in the MINiML format. We have developed a special program that extracts required metadata from MINiML files and provides the user with a choice of possible metadata values. Each GEO dataset was processed using this program. ENCODE provides much more clean and structured metadata that allows us to collect it fully automatically. The raw data in the form of FASTQ files and SRA archives were obtained from the ENCODE and SRA databases, respectively. To avoid variation in results obtained from different ChIP-seq datasets, raw sequenced reads have been processed uniformly by a special workflow.

### Data processing workflow

We used the following special workflow for automatic and uniform processing of collected ChIP-seq data consisting of six steps:

- reduction to a common data format—the FASTQ data format was used for further uniform data processing; ChIP-seq data extracted from SRA in .sra format were converted using the SRA toolkit (http://www.ncbi.nlm.nih.gov/books/NBK158900).
- alignment of reads—we used Bowtie2 (version 2.2.3) (12) to align ChIP-seq reads to the reference human (GRCh38) and mouse (GRCm38) genomes. Bowtie2 is a rather fast and memory-efficient tool able to work with long reference sequences that perfectly suits our needs. Mostly default parameters were used, except that we had a fixed random seed (--seed 0) for reproducibility, used memory mapped I/O (--mm) for more efficient memory utilization, and employed eight threads per bowtie2 process (-p 8). The resulting alignments were converted to .bam files, then sorted and indexed using SAMtools v1.0 (13).
- peak calling—we used four different peak callers to reveal TF binding regions: MACS (14), SISSRs (15), GEM (16) and PICS (17). These four callers were used because they are based on distinct algorithms and take into consideration different aspects of ChIP-seq data. Control experiments were used when available.
- peak clusters—peaks computed for the same TF and peak calling method, but different experimental conditions (e.g. cell line, treatment, etc.) were joined into clusters. Since the width of the peaks reported by different peak callers may vary significantly, we used only peak centers reported by each peak caller and computed the width of binding site based on its assumed width and variation of estimated peak centers. Depending on peak caller, we used different peak centers: for MACS we used the reported ‘summit’ column; GEM reports sites of unit length, and we used this coordinate as the peak center; for PICS and SISSRs, we used the geometric center of the peak. The peaks with centers located 50 bp from each other or closer were merged into one cluster. For each cluster, we found the center by computing the median of the peak centers. We assumed that the width of each cluster should reflect both the actual length of DNA interacting with the protein and uncertainty in the location of TFBSs. As an estimate of this length, the length of the position weight matrix (PWM) for the corresponding TF available from the HOCOMOCO database (http://hocomoco.autosome.ru) was used. When such an estimate was not available, we used the fixed length of 20 bp. The uncertainty in the location of TFBSs was estimated from the variation of peak centers inside that cluster. Specifically, we have computed the standard deviation (SD) of peak centers inside each cluster and used 4*SD as the uncertainty factor in cluster width calculation. When a cluster was supported by only a single peak, the median of SD values from all other clusters was used instead of SD. So, the width of such clusters was computed as the estimated length of DNA interacting with protein + an uncertainty factor.
- metaclusters—clusters for the same TF revealed by different peak calling methods were joined into metaclusters. For this purpose, cluster centers located 50 bp from each other or closer were grouped, and one of them was selected based on priority of peak caller. The priority of peak callers was assigned based on the median SD of peak centers inside clusters in the preliminary analysis. Peak callers showing lower median SD have higher priority. According to analysis results, peak callers were arranged as follows: GEM > PICS > MACS > SISSRs. Metaclusters supported by only one peak caller were filtered out.
- Metaclusters were considered as non-redundant set of TFBSs. Besides information on location in the human or mouse genomes, they contain structured information about cell lines and experimental conditions extracted from the descriptions of corresponding ChIP-seq experiments.
- predicted sites—PWMs from the HOCOMOCO database were also used to predict TFBSs. Further, we plan to use this information for forming clusters and metaclusters. The corresponding algorithm is under development and should be tested for various classes of TFs.

**Figure 1.**
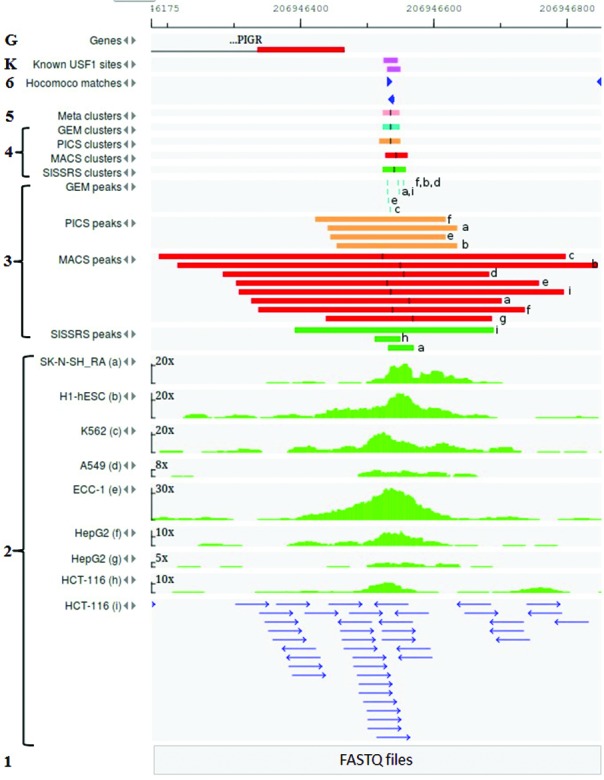
Reconstruction of the human USF1 TFBS in the neighborhood of the PIGR gene by using the GTRD six-step workflow. From the bottom to the top: Step 1: reduction of raw data to FASTQ format; Step 2: read alignment for nine datasets (from a to i; reads of the last one are depicted for demonstration purpose); Step 3: ChIP-seq peaks (with denoted centers) identified by four peak callers for nine datasets a-i; Step 4: peak clusters calculated for each peak caller result; Step 5: metacluster calculated on the base of four clusters; Step 6: USF1 TFBS identified by using respective PWM from the HOCOMOCO database; K. USF1 TFBS known from literature (26,27); G. A part of the PIGR gene structure.

Figure 1 demonstrates steps 1–6 for building of one metacluster for TF USF1.

### Database content and statistics

Supplementary Table S1 summarizes GTRD content and statistics arranged according to the workflow described above.

Most ChIP-seq experiments (61%) have a corresponding control experiment. On average, each TF has been measured in 9.37 ChIP-seq experiments and 291 (∼54%) TFs have been measured in more than one experiment. The most studied TF, CTCF, has been represented by 282 experiments.

### Database maintenance

To maintain GTRD as up-to-date, we have developed a semi-automatic procedure for data mining, processing, accumulation and releasing. A GTRD update is released every 6 months. During this period, new metadata is accumulated automatically or manually from different data sources (GEO, SRA and ENCODE). Finally, new data is automatically processed and merged with the previous release.

### Web interface

**Figure 2.**
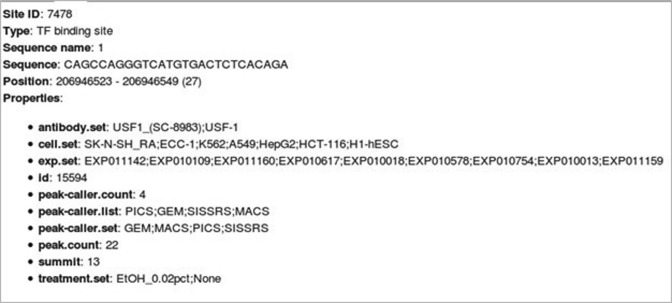
Infocard for the reconstructed USF1 TFBS from Figure 1. Such information is reachable by clicking on a metacluster in the genome browser.

We have developed the GTRD web interface (Figure 2) that provides easy access to data for the most frequent use cases (queries) related to gene expression regulation:

- to find all TFBSs located in regulatory regions of the specified gene;
- to find all the genes that have binding sites for the specified TF;
- to identify cell lines (tissues) and experimental conditions with evidence of binding of the specified TF with a corresponding site;
- to visualize tracks for TFBSs (revealed peaks, clusters, and metaclusters) in the genome browser (Figure 1).

The GTRD landing page (http://gtrd.biouml.org) describes these use cases in detail.

The web interface also provides tools for browsing and displaying information as tables (Supplementary Figures S1 and 2) or as a tree using classification of TF from the TFClass database. It is also possible to download peaks, clusters and metaclusters data in flat files.

The GTRD web interface is developed using the BioUML platform—an open-source integrated Java platform that spans a comprehensive range of capabilities including access to biological databases, tools for visual modelling, parameter fitting and analyses of complex biological systems. It provides powerful possibilities for analyses of high-throughput data with the help of scripting languages (R and JavaScript) and workflows. It also provides a powerful genome browser (18).

We have developed a GTRD plug-in for the BioUML platform that provides necessary analyses:

- searching TFBSs near the specified gene;
- searching genes regulated by the specified TF.

To customize the web interface provided by the BioUML platform for GTRD-specific use cases, we created a GTRD perspective. On the BioUML platform, a perspective is a visual container for a set of views and editors for task-oriented interaction with resources on the platform. Each perspective has a start page with predefined BioUML web components (e.g. specialized forms with input parameters for a data analysis or data query). Like a page within a book, only one perspective is visible at any time. A user can switch between perspectives for solving particular tasks. For example, a user can switch to the HOCOMOCO perspective (http://micro.biouml.org/bioumlweb/hocomoco.html/) for working with the HOCOMOCO database (19) that contains PWMs built on the base of ChIP-seq peaks extracted from the GTRD content. In turn, GTRD contains an experimental track (see step 6 of the GTRD workflow, Figure 1) with TFBSs predicted using PWMs from HOCOMOCO.

## DISCUSSION

Table 1 provides comparison of GTRD with other databases for ChIP-seq experiments.

In the early stages of ChIP-seq technology, the ENCODE project (5) was developed; its aim is to identify all functional genomic elements, and it served as the main source of ChIP-seq data. The ENCODE consortium provides results of data analysis in the form of ChIP-seq peaks for each dataset. As compared with ENCODE, we significantly expanded the collection of ChIP-seq experiments (see database statistics in Table 1) using GEO (10) and SRA (9) databases, and took another step forward in the analysis by merging peaks from different experiments on the same TF into clusters and metaclusters.

Factorbook (20) is based on data from the ENCODE project only and contains a significantly lower number of ChIP-seq experiments.

The CistromeMap/Cistrome DB project (21) achieves high-quality data by manually curating metadata for the large collection of publicly available ChIP-seq experiments. This database uses only one peak caller and does not aggregate peaks from different experiments. We plan to use CistromeMap as an additional source of metainformation in future GTRD releases.

ChIPBase (22) contains significantly fewer ChIP-seq experiments, and these data were not processed uniformly as we do in GTRD. More than 10 different peak callers are used in total, but application of a metacluster approach or similar technique was not observed.

TRANSFAC (23) is a well-known commercial comprehensive database for regulation of gene expression. As of 2016, it contains 23 277 factors, 47 775 TFBSs identified by classical *in vitro* and *in vivo* methods, more than 14 million TFBSs identified by using ChIP technologies and more than 6000 PWMs (https://portal.biobase-international.com/archive/documents/transfacstats.pdf). We could not find exact statistics on the number of ChIP-seq experiments collected there.

ChIP-Atlas (http://chip-atlas.org) has a similar procedure to GTRD for semi-automatic ChIP-seq metadata curation and considers more species than GTRD. However, ChIP-Atlas did not annotate links to corresponding control ChIP-seq experiments that prevents the use of this information in the peak calling procedure. Also, ChIP-Atlas calculates ChIP-seq peaks starting from the raw data and uses a workflow similar to CistromeDB. It provides functions for querying ChIP-seq peaks and target genes similar to GTRD as well as unique functions to search for TF colocalization and enrichment. Unlike GTRD, ChIP-Atlas did not integrate different experiments to provide non-redundant sets of TFBSs.

NGS-QC (24) is a database of quality indicators for the large collection of NGS experiments including ChIP-seq. It has a different purpose than GTRD, but contains similar metadata for ChIP-seq experiments.

GeneProf (25) is a resource of curated, integrated, and reusable high-throughput genomics experiments, including ChIP-seq experiments. Similarly to GTRD, all data were reanalyzed starting from the raw sequencing reads and processed using a consistent workflow. GeneProf contains significantly fewer ChIP-seq experiments than GTRD. And, unlike GTRD, it provides only the results of analyses from individual experiments.

Thus main advantages of GTRD in comparison to other databases for ChIP-seq experiments are the following:

- it contains the most comprehensive (excluding ChIP-Atlas) collection of ChIP-seq data in regards to coverage of different TFs for human and mouse;
- ChIP-seq data were uniformly processed using the workflow described above;
- peaks from different experiments to the same TF were merged into clusters and metaclusters. On the one hand, this allows this information to be made more compact and convenient for a user: on the level of metacluster, the user can see merged results from many ChIP-seq experiments and structured metainformation about these experiments is also available. On the other hand, merging results from many ChIP-seq experiments facilitates more reliable identification of corresponding TFBSs.

The GTRD database is an integral part of a workflow system “From genome to target" which is currently being developed by BIOSOFT.RU, LLC. This workflow system will perform integrated analysis of various omics data and eventually find mechanism-based therapeutic targets and biomarkers referring to the studied disease.

Recently, the ENCODE-DREAM *in vivo* TF Binding Site Prediction Challenge (http://dreamchallenges.org/project/home-open/encode-dream-in-vivo-transcription-factor-binding-site-prediction-challenge/) has been announced. Its main goal is to identify the best-performing model for predicting positional *in vivo* TF binding maps across cell types and tissues. The results of the challenge will also represent a systematic benchmarking and comparison of such computational methods. We plan to use the results of this challenge for further GTRD development.
